# Extreme Heat and Suicide Watch Incidents Among Incarcerated Men

**DOI:** 10.1001/jamanetworkopen.2023.28380

**Published:** 2023-08-11

**Authors:** David H. Cloud, Brie Williams, Regine Haardörfer, Lauren Brinkley-Rubinstein, Hannah L. F. Cooper

**Affiliations:** 1 Department of Behavioral, Social, and Health Education Sciences, Rollins School of Public Health, Emory University, Atlanta, Georgia; 2 Amend at the School of Medicine, University of California, San Francisco School of Medicine; 3 Department of Population Health Sciences, Duke University School of Medicine, Durham, North Carolina

## Abstract

**Importance:**

Extreme heat poses a distinct risk to the 2.1 million incarcerated people in the United States, who have disparately high rates of behavioral health conditions. Suicide is a leading cause of death among people in prisons.

**Objective:**

To examine associations of extreme heat, solitary confinement, and an indicator of suicidal behaviors among incarcerated men in a Deep South US prison system.

**Design, Setting, and Participants:**

This longitudinal case series panel study included adult men in prisons in Louisiana, a state with one of the largest prison systems in the United States that has been engaged in litigation due to lack of air conditioning and extreme heat. The unit of analysis was prison facility-days. A facility-level data set was created by merging administrative data files, which included demographic characteristics, health classification, housing location and movement, disciplinary records, and involvement in suicide-watch incidents for all incarcerated men in Louisiana during the observation period. Individual-level variables were aggregated to facility-days to merge in daily maximum heat index data from the US Local Climatological Data, which were linked to the zip codes of prisons. The observation period was January 1, 2015, to December 31, 2017. Data set construction occurred from August 2020 to September 2022, and analysis was conducted from December 2022 to February 2023.

**Exposure:**

The focal exposure was extreme heat days. Daily maximum heat index data were categorized into 6 bins (<30 °F, 30-39 °F, 40-49 °F, 50-59 °F, 70-79 °F, and ≥80 °F) and as an indicator for any facility-day where the maximum heat index exceeded the 90th percentile of heat indices for total days in observation period. Conditional fixed-effects negative binomial regression models were used to calculate incident rate ratios to test associations between extreme heat and suicide watch incidents, while controlling for covariates.

**Main Outcomes and Measures:**

The focal outcome was daily count of suicide watch incidents that were recorded in a carceral system database. Covariates included daily percentages of incarcerated persons at each prison with serious mental illness diagnosis, daily rate of solitary confinement, and total facility population.

**Results:**

The sample of 6 state-operated prisons provided 6576 facility-days for the analysis. Results suggest a dose-responsive association between extreme heat and daily counts of suicide-watch incidents; compared with days with temperatures between 60 and 69 °F, the rate of daily suicide incidents increased by 29% when the heat index reached the level of caution (ie, 80-89 °F) and by 36% when reaching extreme caution (90-103 °F) (80-89 °F: incidence rate ratio [IRR], 1.29; 95% CI, 1.17-1.43; *P* < .001; 90-103 °F: IRR, 1.36; 95% CI, 1.15-1.61; *P* < .001). Compared with other days, those with the extreme heat indicator were significantly associated with a 30% increase in the incident rate of daily suicide-watch incidents (IRR, 1.30; 95% CI, 1.18-1.45; *P* < .001).

**Conclusions and Relevance:**

Findings suggest an association between extreme heat and an indicator of suicidality among an incarcerated sample, contribute to an emerging literature exploring linkages between climatological events and health outcomes in prisons, and may have implications for legal interventions and advocacy seeking to abate heat-induced morbidity and mortality in carceral contexts.

## Key Points

**Question:**

What is the association between exposures to extreme heat and suicide-watch incidents in a state prison system without air-conditioned living units?

**Findings:**

This case series of 6576 facility-incarceration days found that extreme heat was significantly associated with a 30% increase in the incident rate of daily suicide-watch incidents.

**Meaning:**

These findings suggest that extreme heat may increase vulnerabilities to situations that lead to suicide-watch placements for incarcerated people, bolstering calls for heat mitigation and decarceral interventions to assuage heat-induced harms among incarcerated populations.

## Teaser

This case series examines the association of extreme heat with suicide watch incidents among men incarcerated in facilities without air conditioning in Louisiana.

## Introduction

Extreme heat poses a distinct risk to the 2.1 million incarcerated people in the United States, who have disparately high rates of behavioral health conditions.^1,2,3^ Few jails and prisons have been constructed to endure rising temperatures. Carceral structures are mostly built with materials, such as stone, metal, and concrete, that retain heat and have small or closed windows that impede air circulation, which create conditions for indoor temperatures that exceed those outdoors.^1,4^ Overcrowding is rampant in the US carceral system, with hundreds or thousands of people cramped into poorly ventilated dormitories or small cells (single or double-bunked), which can intensify the physiological and psychological stress of heat exposures.^1,4^ The flip side of extreme overcrowding is extreme isolation (known as solitary confinement), generally defined as being confined in a cell for approximately 22 hours per day, which is defined by international bodies as torture and recognized as a public health and human rights crisis.^5,6^

As recounted in litigation and commentaries, people in solitary confinement are especially susceptible to the hazards of extreme heat because they are less able to avoid or mitigate heat-related stress than those in general population units of prisons or in community settings.^1,4,7^ Yet, only 2 prior studies that we are aware of have explored associations between extreme heat and health among incarcerated persons. A study in Texas prisons^2^ found that an extreme heat day was associated with a 15.1% increased all-cause mortality risk and estimated that approximately 13% of deaths may be attributable to extreme heat in the state’s non–air-conditioned prisons. Another study in Mississippi found that “intensely hot days” were associated with a 20% increased risk of severe violent incidents, and that “unmitigated exposure to heat generates an additional 44 cases of intense violence per year.”^8^

Research has long demonstrated that suicides tend to increase in hotter seasons. In the wake of the climate crisis, recent studies have reported positive associations between higher ambient temperatures and incidence of suicide.^9,10,11^ At a biophysical level, heat stress may worsen mental health symptoms by altering the body’s ability to thermoregulate and regulate emotions.^12^ This may trigger or exacerbate feelings of lethargy, irritability, and sadness, especially for people with existing mental health issues.^12,13,14^ Additionally, studies have linked heat waves and rising temperatures to escalations in community hospitalization rates for behavioral health symptoms, including substance use; mood disorders; schizophrenia and delusional disorders; and nonsuicidal self-harm.^15,16,17,18,19,20,21,22^

People with serious mental illnesses are at increased risk of self-harm and suicide and are also overrepresented in carceral settings.^23,24,25,26^ Living conditions in jails and prisons, including exposures to solitary confinement, can increase vulnerabilities to self-injury and suicide due to exposure to extreme isolation and deprivation.^27^ However, no studies have examined the potential influence of extreme heat on suicidality and its psychological antecedents among people in prison. Therefore, we explored the association between extreme heat, solitary confinement, and suicidal behaviors among incarcerated adult men in a Deep South US prison system.

## Methods

This longitudinal case series panel study merged climatological data with measures of the daily maximum heat index and daily data from the Louisiana Department of Corrections and Rehabilitation (LDCR) (January 1, 2015, to December 31, 2017) and used fixed-effects negative binomial regression models to assess associations between daily exposures to extreme heat and daily incident rate of suicide-watch incidents across 6 Louisiana prison facilities. The unit of analysis was prison-facility days, where facility refers to the 6 prisons in the sample, as described in later sections. A complete consent waiver and Health Insurance Portability and Accountability Act waiver of authorization was approved for this secondary data analysis. This study was approved by the Emory University Institutional Review Board, and data are protected by a federal certificate of confidentiality.

### Setting and Study Sample

Louisiana has one of the largest and most densely populated prison systems in the United States. The state averages 35 days a year when heat exceeds dangerous levels and is projected to experience more frequent, longer, and more severe heat waves, with an average of nearly 115 danger days a year by 2050.^28^ Moreover, LDCR has been embattled in litigation over both solitary confinement and the health-related harms of extreme heat and lack of air conditioning.

### Sampling

The sample for this study included adult men who were in LDCR custody (6 facilities) between January 1, 2015, and December 31, 2017. We used several steps to create a facility-level data set by aggregating individual-level variables to the facility-days based on theoretical and practical considerations. Our sample was limited to people incarcerated in 1 of the 6 state-operated prisons for 75% of the days within the observation period (January 1, 2015, to December 31, 2017).

### Creating the Analytic Data Set

These data were sourced from the Vera Institute of Justice (Vera) and were previously used to produce a report on solitary confinement practices in Louisiana for the Safe Alternatives to Segregation Initiative (SAS-I). These data included raw data files from the Criminal and Justice Unified Network (CAJUN), which is the LCDR’s administrative database that tracks individual-level information on all persons sentenced to imprisonment in Louisiana. These files included demographic characteristics, sentencing, mental health, disciplinary records, housing assignments, and solitary confinement exposures for all persons in Louisiana prisons between January 2015 and December 2017. Access to this data set was obtained through a data-sharing agreement with the Vera Institute of Justice. We created a panel of daily data for each of the 6 state-operated prisons in Louisiana by aggregating individual-level data to facility-level indicators described in more detail in the subsequent sections.

#### Focal Dependent Variable

The focal dependent variable was daily count of newly initiated suicide-watch incidents at the prison-facility level. These incidents were recorded in CAJUN by correctional staff any time a person was placed on suicide watch. A suicide watch occurs when a staff member believes that a person is a potential suicide risk and notifies a supervisor, and a person is placed under observation. These data were obtained at the individual level, which allowed us to determine the date and location (ie, facility) of each suicide-watch incident for each day between January 1, 2015, and December 31, 2017.

#### Focal Independent Variable: Extreme Heat Days

Heat-related data were downloaded from the US Local Climatological Data (LCD), a publicly available resource that tracks hourly, daily, and monthly maximum, minimum, and average temperature, heat index, dew point temperature, relative humidity, degree days (heating and cooling), and daily precipitation. These data are collected from 950 US Automated Surface Observing System stations as well as observations collected every 20 minutes from approximately 1400 US Automated Weather Observing System stations. For this study, we used LCD data on the daily maximum heat index recorded by the weather station linked to the zip code of each of the 6 prisons. Distinct from temperature, heat index is a measure of “what the temperature feels like to the human body when relative humidity is combined with the air temperature”^29^ and is more frequently used to assess the effects of extreme heat on health.^30^ Guided by extant literature,^2,8^ we created 2 indicators of extreme heat. First, we categorized the daily maximum heat index into 6 bins (<30 °F, 30-39 °F, 40-49 °F, 50-59 °F, 70-79 °F, and ≥80 °F) based on the distribution of this variable. For modeling, the reference category was 60 to 69 °F.^8,31,32^ Second, we created a dichotomous indicator for any facility-day where the maximum heat index exceeded the 90th percentile of heat indices for all days in the observation period, based on guidance of prior studies’ definition of extreme heat.^2^

#### Daily Rate of Serious Mental Illness

We aggregated individual-level files to create an indicator of daily percentages of incarcerated persons at each prison classified as levels 1 to 3 as a proxy of serious mental illness (SMI) diagnosis and relative levels of impairment at a facility level.^33^ LDCR uses a level system to classify the acuity and severity of incarcerated persons’ mental health status during the course of their incarceration. It is a time-varying measure, and the date and result of most recent classification is recorded in CAJUN. People classified as level 1 are assessed as having the most severe level of impairment and requiring intensive clinical care, designated housing units, and ongoing management. Those classified as level 2 typically were diagnosed with an SMI and a pattern of functional instability within the past 6 months. People classified as level 3 have an SMI diagnosis but have been stable on medication and functionality measures for at least 6 months. People on level 4 typically have an axis I (according to the *Diagnostic and Statistical Manual of Mental Disorders* [Fourth Edition]) diagnosis other than SMI and a history of substance dependency.

#### Daily Rate of Solitary Confinement

We aggregated individual housing files to calculate the total number of incarcerated persons residing in solitary confinement each day and then divided it by the total population at each prison-facility that day. A person was counted as being in solitary confinement on a particular day if housing records showed they were assigned to 1 of the following types of units, designated by location codes in CAJUN: administrative segregation, extended lockdown, closed-cell restriction, or death row.

#### Daily Facility Population

Daily facility population was defined as the total number of people incarcerated at each prison on a given day divided by Louisiana’s total imprisoned population. This control variable was calculated using housing files to count the unique individuals in each prison for every day in the observation period.

### Statistical Analysis

There were very minimal missing data in our data set, and only for the solitary confinement variable. The Vera Institute of Justice data set from which solitary confinement exposures were obtained only went from January 1, 2015, to July 3, 2017. Therefore, we had missing data on facility-level rates of solitary confinement for approximately 15% of the 6576 facility-incarceration days. These data were missing completely because Vera did not have solitary confinement data for the last 6 months of 2017 and were not influenced by the presence or absence of other variables in our data.

First, we conducted descriptive analysis to explore facility-level variations in suicide-watch incidents, extreme heat, solitary confinement, and mental health severity over time. We confirmed the accuracy of our data merging procedures by comparing results of our descriptive analysis with those in other sources.^33,34^ Next, we conducted bivariate analysis of each theoretically relevant factor prior to running full models.

For robustness, we compared results from 3 different approaches to handling missing solitary confinement data. First, we ran the models with the data as missing (ie, excluded daily rate of solitary confinement at each facility for roughly 6 months). We also performed multiple imputation by including all factors in the model, running 500 iterations.^35^ As discussed later, each approach had comparable results.

**Figure 1.**
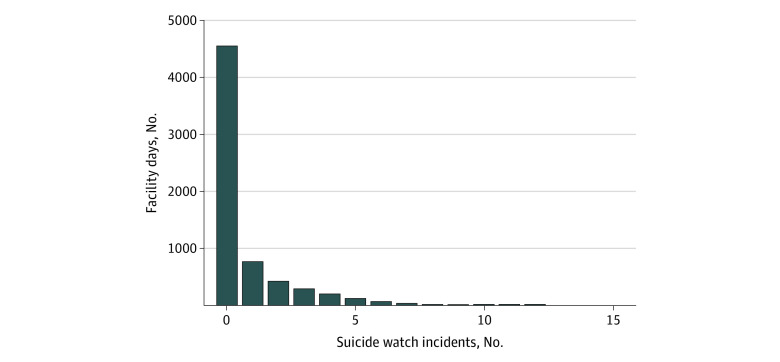
Distribution of Daily Counts of Recorded Suicide Watch Incidents Figure 1 shows the distribution of the daily counts of suicide watch incidents recorded in the administrative records of the Louisiana Department of Corrections and Rehabilitation between January 2015 and December 2017.

Since our focal outcome was a count (ie, daily frequencies of suicide-watch incidents) that was not normally distributed (Figure 1) and overdispersed (variance exceeded the mean value), we used conditional fixed-effects negative binomial regression models to test associations between extreme heat and suicide-watch incidents, while controlling for aforementioned covariates.^35,36^ We included fixed effects for day and facility in the model to account for clustering and potential influence of variations in unobserved factors over time and facility. We also added a fixed effect for month to our models to account for potential seasonality effects that may influence the incident rate of suicide-watch incidents. Extreme heat is an exposure that is independent of facility-level exposures that may shape the incidence rates of suicide watches within and between prisons, especially when considering the ubiquitous absence of air conditioning in the sampled prisons during the observation period. Conditional fixed-effects negative binomial regression models were performed using XTNBREG commands in Stata version 17 (StataCorp). We ran 2 models to assess the association between extreme heat and suicide watch incidents: one using the heat-index-bin indicator (60-69 °F as reference group) and a second model using the binary indicator for facility-days exceeding the 90th percentile of heat-index from January 1, 2015, through December 31, 2017. The data analysis was conducted from December 2022 to February 2023. Statistical significance was set at *P* < .05.

## Results

**Figure 2.**
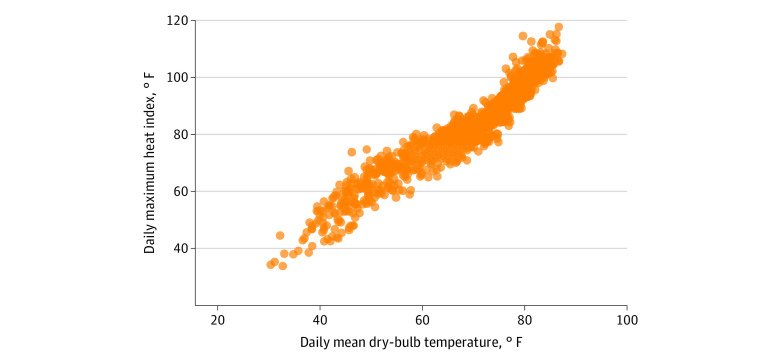
Estimated Maximum Daily Heat Index for Total Sample, 2015 to 2017 Figure 2 displays the distribution of the maximum heat index for each facility-day among the prisons in the total sample. These are plotted by heat index bins.

The sample included 6 state-operated prisons in Louisiana, with a total of 6576 facility-days. The average daily population and demographic composition of each facility are reported in Table 1. Across all prisons, the mean (SD) daily number of suicide-watch events was 0.79 (1.56) (Figure 1). Each facility experienced similar percentage of days (mean [SD] days, 108 [0.29] days; 9.8%) during the observation period that met the definition of an extreme heat day (ie, exceeded the 90th percentile maximum daily heat index) with the exception of Raymond Laborde Correctional Center, which had 152 days (13.9%) in the extreme heat range for the observation period (Figure 2). The mean (SD) daily maximum heat index across all prisons was 84.5 (17.1) °F with little variation between prisons (Table 1). Across all 6 prisons, the mean (SD) average percentage of people in solitary confinement was 21.2% (11.6), although it varied by facility and ranged from 6.9% to 28.9%. The estimated daily mean (SD) population with an SMI and classified as having more severe functional impairment (ie, level 1-3 mental health status) varied by facility as follows: Louisiana State Penitentiary, 10.2% (1.1); Elayn Hunt Correctional Center, 17.4% (1.1); Raymond Laborde Correctional Center, 14.5% (1.6); Rayburn Correctional Center, 10.3% (1.1); David Wade Correctional Center, 9.9% (1.0); and Dixon Correctional Center, 5.8% (0.9). In bivariate analysis, all putative factors reached statistical significance and were included in the final models (Table 2).

**Table 1.** Descriptive Statistics for Facility-Level Variables for 6576 Facility-Days, January 1, 2015, to July 3, 2017

Characteristic	Prison facility					
	LSP	EHCC	DWCC	DCC	RLCC	RCC
Daily facility population, mean (SD), No.	4200 (117.6)	1330 (23.0)	854 (14.5)	1259 (16.2)	1273 (17.4)	940 (6.7)
Total count of suicide watch incidents, No.	1561	3236	109	46	29	195
Daily count of all suicide watch incidents, mean (SD), No.	1.5 (1.7)	3.1 (2.3)	1.0 (0.4)	0.7 (0.3)	0.6 (0.2)	0.2 (0.5)
Facility-days with a suicide watch incident, %	66.2	89.5	8.9	4.0	2.5	15.9
Daily maximum heat index, mean (SD), °F	84.3 (17.7)	84.8 (16.3)	82.7 (18.3)	84.6 (16.5)	85.7 (17.7)	84.8 (16.3)
Facility-days at extreme heat, %	9.0	9.4	8.3	8.9	13.9	8.9
Daily percentage of population in solitary confinement, mean (SD), %	25.8 (5.2)	30.2%(6.4)	35.8 (7.0)	7.9 (3.1)	10.0 (4.4)	17.8 (5.3)
Daily percentage of population with serious mental illness, mean (SD), %	10.2 (1.1)	17.4 (1.1)	9.9 (1.0)	5.8 (0.9)	14.5 (1.6)	10.3 (1.1)

Abbreviations: DCC, Dixon Correctional Center; DWCC, David Wade Correctional Center; EHCC, Elayn Hunt Correctional Center; LSP, Louisiana State Penitentiary; RCC, Rayburn Correctional Center; RLCC, Raymond Laborde Correctional Center.

**Table 2.** Bivariate Associations With Suicide Watch Incidents Using Conditional Negative Binomial Regression With Fixed Effects

Variable	Incident rate ratio (95% CI)^a^
Heat index bins	
21-29 °F	0.24 (0.11-0.52)
30-39 °F	0.83 (0.78-0.87)
40-49 °F	0.86 (0.84-0.89)
50-59 °F	0.69 (0.66-0.71)
60-69 °F	1 [Reference]
70-79 °F	1.07 (1.05-1.12)
80-89 °F	1.43 (1.39-1.46)
90-103 °F	1.33 (1.26-1.40)
Extreme heat day	1.37 (1.33-1.40)
Daily percentage of population in solitary confinement	1.01 (1.00-1.02)
Daily percentage of population with serious mental impairment	1.04 (1.03-1.04)
Daily facility population	0.99 (0.99-0.99)

^a^ All variables reached statistical significance at *P* < .05.

Results of the multivariable model suggested a strong and dose-responsive association between extreme heat and the daily incident rate of suicide watches across analyses (Table 3). Within the hotter daily heat index bins (ie, those exceeding 60-69 °F), the incidence rate of daily suicide incidents increased by 29% when the heat index reached the level of caution (ie, 80-89 °F) and by 36% when reaching extreme caution (90-103 °F) (80-89 °F: incidence rate ratio [IRR], 1.29; 95% CI, 1.17-1.43; *P* < .001; 90-103 °F: IRR, 1.36; 95% CI, 1.15-1.61; *P* < .001). Moreover, while the cooler heat-index bins (ie, those below the reference group of 60-69 °F) were not statistically significant, the model indicates that cooler days may decrease the incidence rate of suicide-watch incidents. Results of model 2 corroborated model 1 and suggested that compared with other days, those falling into the extreme heat category were significantly associated with a 30% increase in the incident rate of daily suicide-watch incidents (IRR, 1.30; 95% CI, 1.18-1.45; *P* < .001). In other words, extreme heat days contained 30% more suicide-watch incidents than days below that range.

**Table 3.** Significant Factors for Daily Suicide Watch Incidents in 6 Louisiana State Operated Prisons, 2015 to 2017^a^

Variable	IRR (95% CI)	*P* value
**Model 1**		
Heat index bins (reference group 60-69 °F)		
21-29 °F	0.72 (0.34-1.52)	.39
30-39 °F	0.77 (0.59-1.00)	.05
40-49 °F	0.89 (0.77-1.05)	.17
50-59 °F	0.92 (0.81-1.05)	.21
60-69 °F	1 [Reference]	NA
70-79 °F	1.08 (0.98-1.19)	.14
80-89 °F	1.29 (1.17-1.43)	<.001
90-103 °F	1.36 (1.15-1.61)	<.001
Percentage of population in solitary confinement	1.01 (1.00-1.02)	.02
Percentage of population with serious mental illness	1.06 (1.04-1.07)	<.001
Daily facility population	1.23 (0.200-7.535)	.83
**Model 2**		
Extreme heat day	1.30 (1.18-1.45)	<.001
Percentage of population in solitary confinement	1.01 (0.92-1.02)	.06
Percentage of population with serious mental illness	1.06 (1.10-1.80)	<.001
Total daily facility population	1.39 (0.87-8.12)	.71

Abbreviations: IRR, incidence rate ratio; NA, not applicable.

^a^ Final model only included variables whose parameter estimates were significant at the *P* < .05 level. Model included fixed effects for day and prison facility. Extreme heat day refers to any facility-day that exceeded the 90th percentile of the daily heat index for the total observation period.

Both models suggest facility-level indicators of solitary confinement and level of mental health need were significantly associated with suicide-watch incidents. First, both models indicate that 1-SD increase in the percentage of people held in solitary confinement was associated with a 1.0% increase in the incident rate of daily suicide-watch incidents. These models also suggest that for each SD increase in the daily percentage of incarcerated people classified as having a higher level of mental health need (ie, level 1-3 mental health status), the incident rate of daily suicide-watch incidents increases by 6.0%, a finding that is corroborated in model 2 (Table 3).

## Discussion

We studied the associations of extreme heat, solitary confinement, and suicidal behaviors among incarcerated adult men in Louisiana. According to our findings, concerns about the perils of heat exposures for incarcerated populations are well-founded: the incident rate of suicide watches was 29% greater on days when the maximum heat index reached 80 to 89 °F and 36% greater on days climbing into the 90 to 103 °F range, after controlling for relevant facility-level covariates and potential seasonality effects.

To our knowledge, this is the first study to link extreme heat and an indicator of suicidality among a sample of imprisoned people. Our observations align with evidence from prior studies linking extreme heat to increases in psychiatric morbidity, utilization of clinical protocols for mental health emergencies, and incidence of suicidality in other contexts.^11,15,37,38,39^ This study expands on theory and empirical evidence showing a positive association between heat exposures and incidence of different forms of violence. Similar to our findings, Mukherjee et al^8^ observed an association of heat with daily counts of violent assaults in the Mississippi prison system as the heat index exceeded the 80 °F range. Echoing others, Colucci et al conceptualized the hazards of extreme heat in carceral spaces as form of “thermal (in)equity”^1^ and urged geographers, epidemiologists, and environmental scientists to forge partnerships to advance science and formulate intersectoral interventions for preventing and redressing associated harms. Our methods, alongside previous studies, provide insights into the promises of merging climatological, carceral, and other sources of data to carry out such studies and visualize hot spots for emergent environmental injustices.^40^

In response to extreme heat, prisons often implement strategies such as flagging people who are vulnerable and providing access to fans, ice, and cold showers.^7^ However, these policies are typically intended to avert heat exhaustion or heat stroke and dehydration and have been less attuned to the psychological and behavioral effects of extreme heat. However, more systemic change is needed. Our results may help amplify the need for systematic changes. Most US prisons and jails do not have air conditioning. Carceral systems in settings that expect to be most impacted by climate change (eg, the Southeast) should allocate funds to update buildings accordingly.^7^ Additionally, due to the excess harms prison conditions can impose in the context of excess heat, policy makers should heed to calls to pursue decarceration and anticarceral solutions as environmental justice and public health imperatives.^41,42,43^

### Limitations

There are several limitations in this study. Drawing on administrative data likely underestimated magnitudes of suicidality and self-injury in these prisons and did not permit accounting for differences in how extreme heat may influence suicidality vulnerabilities in spaces used for solitary confinement vs dormitories and other types of units. Additionally, our measurement of heat index recorded outdoor exposures and therefore could not account for variations in indoor heat index exposures within and between facilities. However, aside from several cells in a death row unit, and 1 other tier in 1 prison, none of the living spaces in the 6 prisons was air-conditioned.^33^ As a result, it is likely that the heat index inside prison cells exceeded the outdoor measure due to the physical infrastructure, as documented in recent litigation.^7,44^ The generalizability of this study is limited because the sample only included incarcerated adult men: incarcerated women were displaced after the only prison designated for women in Louisiana was evacuated during the study period, which limited the sample to adult men. Nationally, self-injury and suicide are highly prevalent among incarcerated women; therefore, studies that better account for gender and variations in geography and temperature control capacities are needed.^27^ Similarly, in our facility level analysis, we did not have reliable data on race and ethnicity and were unable to determine whether anyone in the facility died by suicide. Additionally, due to the small number of prisons in our sample, hierarchical modeling techniques, which are better suited for accounting for the influence of between- and within-facility variations in exposures and covariates on the focal association, where not possible. Increasing the number of facilities, enhancing measurement instrumentation, including additional data sets, and expanding the observation period are important goals for subsequent studies on heat and health in carceral settings.

## Conclusions

As the warming of the planet escalates and the US continues to incarcerate more of its population than any sovereignty in modern history, a host of humanitarian and public health emergencies are likely to unfold. This study illuminates the connection between solitary confinement, extreme heat, and self-injury. It offers evidence to enhance the public health rationale for urgent calls for air conditioning and other heat-mitigation protocols in carceral spaces in the short-term and underscores the importance of seeking long-term solutions through collective movement building in pursuit of environmental justice, human rights, and the abolition of carceral spaces.

## Notes

Supplement.
Data Sharing Statement
