# Supplementary material for: Extreme Heat and Suicide Watch Incidents Among Incarcerated Men
Source: JAMA Netw Open. 2023 Aug 11;6(8):e2328380. doi: 10.1001/jamanetworkopen.2023.28380 (PMC10422184; doi:10.1001/jamanetworkopen.2023.28380)
Supplement: Supplement. — Data Sharing Statement [file jamanetwopen-e2328380-s001.pdf]

Data Sharing Statement

Cloud. Extreme Heat and Suicide Watch Incidents Among Incarcerated Men. *JAMA Netw Open*. Published August 11, 2023. doi:10.1001/jamanetworkopen.2023.28380

Data
Data available: No

Additional Information
Explanation for why data not available: The dataset used for this study cannot be shared per terms of a memorandum of understanding.
